# Nearby grandmother enhances calf survival and reproduction in Asian elephants

**DOI:** 10.1038/srep27213

**Published:** 2016-06-10

**Authors:** Mirkka Lahdenperä, Khyne U. Mar, Virpi Lummaa

**Affiliations:** 1 Section of Ecology, Department of Biology, University of Turku, FIN-20014 Turku, Finland; 2 Department of Animal and Plant Sciences, University of Sheffield, Sheffield S10 2TN, UK

## Abstract

Usually animals reproduce into old age, but a few species such as humans and killer whales can live decades after their last reproduction. The grandmother hypothesis proposes that such life-history evolved through older females switching to invest in their existing (grand)offspring, thereby increasing their inclusive fitness and selection for post-reproductive lifespan. However, positive grandmother effects are also found in non-menopausal taxa, but evidence of their associated fitness effects is rare and only a few tests of the hypothesis in such species exist. Here we investigate the grandmother effects in Asian elephants. Using a multigenerational demographic dataset on semi-captive elephants in Myanmar, we found that grandcalves from young mothers (<20 years) had 8 times lower mortality risk if the grandmother resided with her grandcalf compared to grandmothers residing elsewhere. Resident grandmothers also decreased their daughters’ inter-birth intervals by one year. In contrast to the hypothesis predictions, the grandmother’s own reproductive status did not modify such grandmother benefits. That elephant grandmothers increased their inclusive fitness by enhancing their daughter’s reproductive rate and success irrespective of their own reproductive status suggests that fitness-enhancing grandmaternal effects are widespread, and challenge the view that grandmother effects alone select for menopause coupled with long post-reproductive lifespan.

Humans, killer whales (*Orcinus orca*) and short-finned pilot whales (*Globicephala macrorhyncus*) have an exceptional life-history, with a notable proportion of all females in a given population post-reproductive and one quarter to half of the long lifespan spent as post-reproductive^1^,^2^,^3^. The “grandmother hypothesis”^4^ has been suggested as the adaptive mechanism resulting in the evolution of such post-reproductive longevity. Mothers that improve the success of their adult offspring by providing prolonged care and help beyond the end of their own fertility may contribute more grandoffspring into the next generation, imposing selection for a long post-reproductive lifespan. In support of this, accumulating evidence shows that helping post-menopausal women are able to increase the survival and reproductive capacity of their own offspring as well as the survival of their grandoffspring^5^,^6^,^7^. Recent studies also suggests that similar to humans, post-reproductive killer whale females can gain adaptive benefits through improving their adult sons’ survival^8^ and grandmothers have positive effects on 3-year old calf survival^9^.

However, a recent review of the current evidence for the grandmother hypothesis highlighted the need to scrutinize its predictions more widely in species that do not exhibit verified menopause and long post-reproductive lifespans, to provide a comparison and to further our understanding of the evolution and selection pressures on post-reproductive lifespan and long life in general^2^. Indeed, several species without menopause also show evidence of grandmaternal help but the potential fitness benefit of this behavior remains unclear. For example, aging females are known to show various behaviors beneficial to their descendants ranging from group protection to direct care in many primates, birds, cetaceans and even invertebrates^10^,^11^,^12^,^13^,^14^. However, the genetic relationship of the older females to the younger individuals is often unknown, and thus little can be concluded with certainty about the fitness effects of grandmothers. Also, while the evidence of singular cases of a helping grandmother is large^15^,^16^,^17^, the evidence of measurable fitness effects of grandmothers in non-menopausal taxa comes from only a few species. The presence of grandmothers increased the survival of grandoffspring in vervet monkeys (*Chlorocebus aethiops sabaeus*)^18^ and Japanese macaques (*Macaca fuscata*)^19^. In addition, grandmothers enhanced the reproductive rate of their daughters in both species^19^,^20^. However, although about 60% of the daughters had a living mother at the time of reproducing^18^,^19^, the presence of post-reproductive grandmothers in these species was short-term and available for only a few grandoffspring^19^, and none or only a minority (2.8%) of the grandmothers had terminated reproduction themselves^18^,^19^. It has also been speculated that in many cetaceans for which the data is limited, older females, possibly grandmothers, act as allomothers and even lactate the grandcalves^21^,^22^,^23^. In lions (*Panthera leo*)^24^, reproductive grandmothers increased the survival of their grandoffspring, potentially because of allolactation, but had no effect on their daughter’s litter size. Importantly, grandmothers with heavy own current reproductive costs are predicted to invest less in their grandoffspring than post-reproductive grandmothers without such costs^19^,^25^, but whether the detected grandmaternal effects are greater for grandmothers which have ceased reproduction and whether these helping females are particularly long-lived compared to other females in line with the predictions of the grandmother hypothesis are largely unknown. These questions are in pivotal role to unravel the selection pressures on post-reproductive lifespan and to elucidate whether the fitness benefits of grandmaternal effects alone lead to the evolution of menopause, or whether such effects are widespread in social species with and without menopause and extended post-reproductive lifespan.

Elephants provide a particularly promising opportunity to test the hypothesis by investigating several of its key predictions. Well-known for their close relationships between females within multi-generational herds led by matriarchs^26^,^27^, elephants have no clear-cut menopause and females are capable of reproducing beyond 60 years^3^,^28^. They are long-lived, with a maximum lifespan reaching 80 years. Although elephants lack menopause, Asian elephant (*Elephas maximus*) females which survive to old age can have relatively long lives after last reproduction, at the population level, 17 years after age 47 (when 95% of population fecundity has been realized) and at the individual level 11 years for those females living beyond 40 years^3^. Similarly, African elephant (*Loxodonta africana*) females live 16 years on average after age 49^29^. Increasing evidence supports the importance of older females for group wellbeing/survival^27^,^30^,^31^, allomothering/grandmothering among elephant groups^29^,^32^,^33^,^34^,^35^ and recent evidence shows enhanced reproduction of daughters with longer living mothers in African elephants^29^. However, specific quantitative data testing the grandmother hypothesis in elephants or other long-lived species without clear menopause is still lacking, leaving it untested whether the post-reproductive or long lifespan in itself could be explained by the helping behavior of grandmothers.

Here we test the key predictions of the grandmother hypothesis by taking advantage of multigenerational longitudinal data recorded for Asian elephants working in timber extraction locations in Myanmar. The dataset provides a good opportunity for the following reasons: the population is semi-captive but with free interaction outside working hours, social groups roam in the forest unsupervised during free time, mortality rates are close to wild elephants^36^, and reproductive rates are not influenced by humans (humans do not intervene or help with matings/ the calving process and many calves are thought to be sired by wild bulls). In detail, we test this hypothesis by investigating first the effects of (grand)mother presence on the reproductive success of their daughters by comparing whether a living grandmother residing and working in the same vs. another logging location as the grandoffspring (at the time of the birth) has an effect on the grandoffspring survival until their weaning age at 5 years. Second, we investigate the effects of grandmothers on their daughter’s reproductive rate by comparing whether grandmother presence affected the length of daughter’s inter-birth intervals, contrasting grandmothers residing in the same vs. another logging location. Third, we test whether the grandmother’s reproductive status has an effect on the grandoffspring survival. In all analyses we also investigate the effect of grandmaternal age to find out whether older and younger grandmothers differ in their investment in their (grand)offspring. We use a dataset including 161 calves (from 95 mothers and 83 grandmothers) which had living grandmothers at the time of their birth, of which 112 had a grandmother living in another location and 49 in the same logging location. Within a given logging location, grandmothers, daughters and grandoffspring had an opportunity to interact during non-working hours. We control for larger-scale geographic variation in demographic rates across Myanmar, varying time trends during our 35-year study, between-grandmother differences as well as several variables accounting for individual differences in calf mortality and inter-birth intervals. This study provides a much needed comparison of a long-lived non-menopausal terrestrial mammal with kin-based social groups to species with menopause and decades long post-reproductive survival, as these traits are suggested to have evolved through fitness enhancing grandmother effects.

## Results

**Figure 1:**
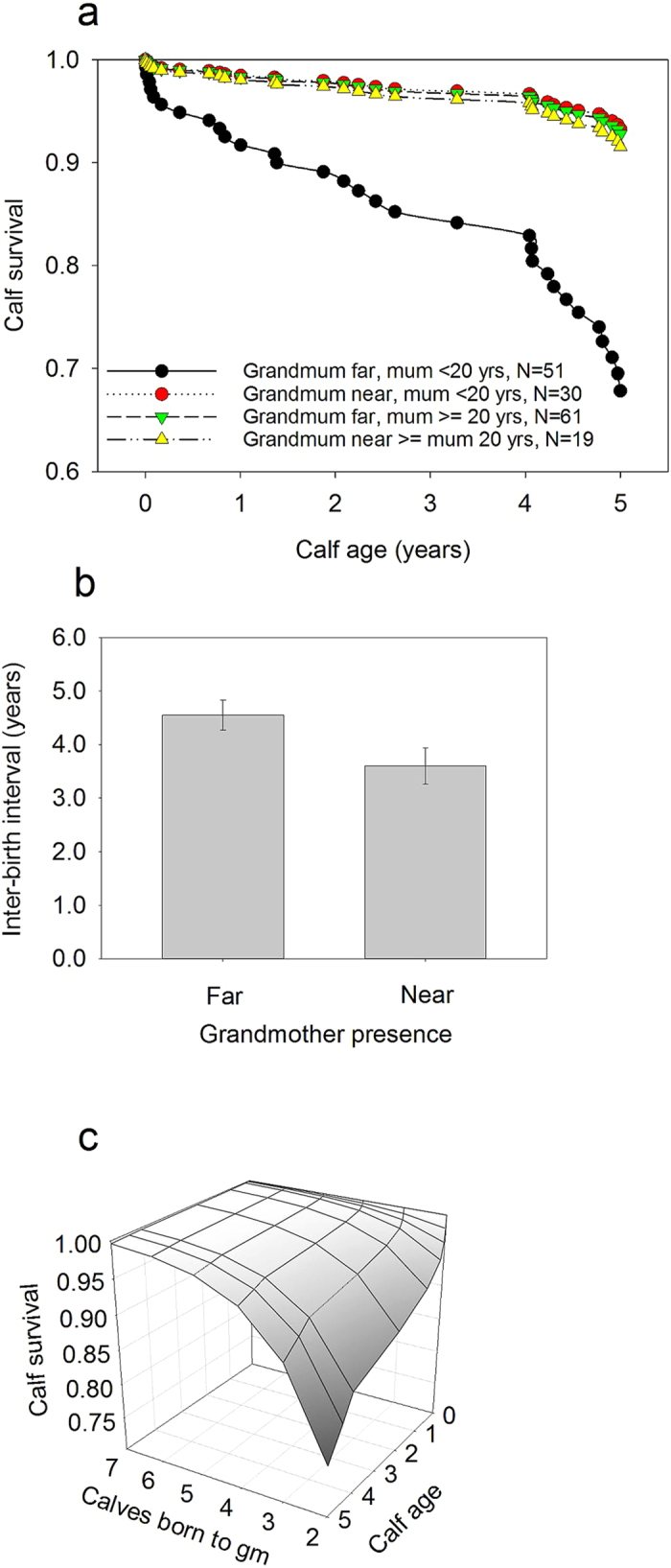
Grandmother proximity, calf survival and inter-birth intervals. (**a**) Calves born to young mothers and residing in the same logging location as their grandmothers and able to interact with her (“near”) had significantly higher survival than calves residing in different location to their grandmothers (“far”). (**b**) Calves were also born with shorter inter-birth intervals if the grandmother resided in the same location compared to when she lived elsewhere. (**c**) The more offspring the grandmother had given birth to before the grandcalf, the higher was the grandcalf survival to 5 years. Drawn according to reference categories of controlled variables in the final model and without a random term in the Cox model (**a,c**). (**b**) Shows standard errors (S.E) and predicted values. In (**c**) gm refers to grandmother.

Overall, 23% of calves died before reaching 5 years. Only 6 of the 161 calves lost a mother before age 5 years and all of the calves in the sample had a living grandmother at the time of their birth. After controlling for all fixed and random terms, we found that having a grandmother residing in the same location as the grandcalf significantly decreased calf mortality, but the strength of this effect varied depending on the age of the calf’s mother (Table 1; χ_*1*_^2^ = 4.85, Adjusted p = 0.019). If the mother was younger than average (20 years) when the calf was born and the grandmother resided in another location, the calf had an 8 times greater risk of dying than if the grandmother was present in the same logging location (Hazard ratio (95% CLs): 8.40 (1.13, 62.30); pairwise comparison: χ_*1*_^2^ = 4.33, p = 0.037; Fig. 1a). However, if the mother was older than 20 years when the calf was born, the grandmother presence did not affect calf mortality (Hazard ratio (95% CLs): 0.59 (0.15, 2.34); pairwise comparison: χ_*1*_^2^ = 0.57, p = 0.45; Fig. 1a). The calf mortality was also modified by calf sex and maternal age as female calves had higher mortality when the mother was younger than average and male calves slightly higher when the mother was older than average (Table 1). Finally, there was significant variation in calf mortality between birth cohorts, origin and different regions across the country, and between different grandmothers (Table 1).

Grandmother’s presence in the same location had beneficial effects also on shortening the daughter’s inter-birth intervals. If the grandmother was alive and in the same location with the daughter’s two subsequent calves, the birth interval of these calves was 0.95 years (21%) shorter than if the grandmother resided in a different location (Fig. 1b; Table 2; *F*_1,68_ = 6.73, p = 0.012). This beneficial effect of grandmothers was similar irrespective of maternal age, so the birth rate of all females was improved by grandmother presence. Inter-birth intervals were also generally significantly decreased if the previous calf died before reaching age 2 (Table 2; survived vs. not respectively: 4.57 ± 0.23 yrs. vs. 3.58 ± 0.40 yrs.), for female calves, young mothers and later born calves of young mothers; our models adjust for these differences (Table 2). Such effects of (grand)mothers on their daughter’s interbirth intervals were of importance, because grandmothers known to be resident alongside at least one young (<20 years) daughter had significantly higher total number of grandoffspring born as compared to females which were not resident alongside any of their daughters, or were only resident alongside their older (>20) daughters Est. (gm resident with young daughter) = 0.30 ± 0.14; (*F*_1,76_ = 4.64, p = 0.035; 4.43 ± 0.44 vs. 3.28 ± 0.32 respectively; see Methods).

To further investigate the predictions of the hypothesis and the mechanisms in our study population in Myanmar, we determined whether the grandmother’s reproductive status or previous number of calves at the time the grandcalf was born affected her grandcalf mortality. The grandmother’s reproductive status (whether or not the grandmother reproduced within 3 years of the birth of the grandcalf) did not have an effect on the grandcalf mortality (Table 3; χ_*1*_^2^ = 0.49, *p* = 0.48; Hazard ratio (95% CLs)(reproducing gm): 1.70 (0.39, 7.53)). However, the more offspring the grandmother had given birth to before the grandcalf was born, the lower the mortality of the grandcalf (range: 2–7 calves; Fig. 1c; Table 3; χ_*1*_^2^ = 4.81, *p* = 0.028). Calf mortality was also affected by the maternal age (Table 3).

## Discussion

Inter-generational help and the investment of older generations into the young^37^ are important for highly social, long-lived species such as humans and killer whales, and such investments have been suggested to be the evolutionary reason underlying the long post-reproductive lifespan in these species^2^,^4^. However, some evidence from other species suggests that grandmaternal effects might also exist among non-menopausal taxa. We measured the effects of grandmothers in one of the longest living terrestrial species, the Asian elephant. We found that grandmother’s presence was highly beneficial for grandcalf survival particularly among young mothers and the daughters were also able to reproduce more rapidly in the presence of their mothers in a semi-captive population of elephants in Myanmar. Such findings have implications for our understanding of the evolution and selection pressures on post-reproductive lifespan in general, as well as on dispersal and social system in elephants specifically.

Although females in elephant groups are usually closely related to each other and singular cases of helping grandmothers have been observed in both African and Asian elephants in previous studies^32^,^33^,^38^, little has been known about the fitness effects of grandmothers per se (but see^29^). Our results show for the first time that Asian elephant grandmothers, irrespective of their age or status, have a significant positive effect on the reproductive success of their offspring. The results are in line with another study of African elephants in Amboseli where grandmothers increased their daughter’s first calf survival^35^. In this study the grandmother presence was not directly measured at the time the grandcalf was born but, rather, grandmothers were assumed to be present in the family unit if they were known to have survived until the daughter was 15 years old. The study showed that 18% of first calves died if the grandmother was alive in comparison to a 31.5% average for calves of primiparous females. We found even higher impact of grandmothers in our population of Asian elephants as 32% of young mothers’ calves died before reaching age 5 years if the grandmother was in a different location than themselves and only 7% died in the presence of the grandmother. Although the majority of these calves were firstborn to their mothers (65% of the calves in the same location with their grandmothers and 52% of the calves in different location) we found that maternal age had more significant effect on calf survival than the calf’s birth order. The positive effect of grandmothers was not detected if the mother was older than 20 years at the time the calf was born. Interestingly, in addition to elephants, vervet monkey grandmothers have also been shown to increase their grandoffspring survival^18^, and in this species grandmothers spent more time with their younger daughters’ infant if they had more than one adult daughter present^39^. Although in our study the elephant grandmothers rarely had the option to choose who to help because most only had one reproductive daughter simultaneously co-residing with them, a general picture emerges where grandmothers may be particularly important for the reproductive success of their inexperienced adult daughters. Such need of younger generations to learn the mothering skills from the older generation may be one potential mechanism resulting in higher fitness through grandmothering effects.

We also found that grandmother presence shortened the inter-birth intervals between the daughter’s subsequent calves. The one year decrease in birth interval length is notable as early mortality of the previous calf decreases birth-intervals to a similar magnitude but unlike grandmother effects, is well-known in previous literature for decreasing birth intervals in elephants by allowing females to resume cycling sooner^40^. Furthermore, the birth rate of all females was improved by grandmother presence, irrespective of the maternal age. Interestingly, in African elephants, longer presence of a mother was also correlated with increased age-specific rate of reproduction of a daughter^29^, suggesting that similarly to humans^7^, grandmothers may generally improve both daughter reproductive success (offspring survival) and reproductive rate and both of these effects should be considered simultaneously when evaluating the overall fitness effects of grandmothers in different species.

That grandmother presence was associated with clear benefits to daughter’s reproductive success brings up the question; how does the grandmother presence lead to these benefits? First, the grandmother could shorten the daughter’s inter-birth intervals by lightening the daughter’s required investment in the current offspring^41^, resulting in younger weaning age and earlier reproductive cycling^42^. Second, the grandmother could provide additional help to the daughter, which increases their offspring survival^41^. The grandmother hypothesis predicts that the central mechanism among humans has been food sharing by grandmothers with weaned grandoffspring^4^. In Asian elephants, allolactation has been commonly observed but it is currently unknown how often grandmothers, specifically, engage in this behavior^33^,^38^. Opportunity for social bonds with relatives may also benefit mothers per se, because close, long-lasting social bonds among females are known to have beneficial effects also on primate reproductive success and longevity^43^,^44^.

The grandmother hypothesis also predicts that reproductive grandmothers cannot invest as much in their grandoffspring as post-reproductive grandmothers^19^,^25^. In line with this, in Asian elephants, some observations suggest that mothers with suckling calves never permitted other calves to suckle them whereas allolactation was more common among females which had currently no calf of their own^33^,^38^. However, in non-menopausal taxa reproduction might also have an opposite effect: breeding lion grandmothers increased the survival of their grandoffspring, potentially because of allolactation^24^. We found that the grandmother’s reproductive status (whether or not the grandmother reproduced within 3 years of the birth of the grandcalf) had no effect on grandcalf survival. This suggests that whatever the mechanism involved, the grandmother’s own current reproductive costs do not hinder her ability to provide grandmaternal benefits simultaneously to her grandoffspring. However, our findings do not rule out also the possibility of allolactation by the grandmother.

That the grandmother’s breeding status or age did not modify the benefits her presence incurred to her daughter’s reproductive success is surprising in the light of previous studies, and more importantly, contrasts with the predictions of the grandmother hypothesis. Age is correlated with dominance in elephants^35^,^45^, which is known to affect group fitness and survival^27^,^30^,^46^. Although we were able to control for grandmother age in our models, accurate dominance status of the historical grandmothers included in this study (many of which are now deceased) is unknown. That such age effects were absent is of interest in the light that in African elephants, too, the grandmother presence was observed to be more important for the survival of the (grand)offspring than the grandmother’s social status per se: having a matriarch as a mother did not have an effect on daughter’s first born calf survival^35^. However, the reason that we were unable to find any effect of grandmaternal age might be the relatively small sample size of very young grandmothers (10% <33 years old), which are likely to be the least experienced and not as beneficial to their offspring as the older grandmothers. Alternatively, grandmothers of any age increased the daughter’s reproductive success but the eldest females can still have some fundamentally differing skills benefitting the whole group. The benefits of knowledge of the older generation is seen in some other long-lived species such as killer whales, where the post-reproductive females are known to lead group movements especially during years of lower prey abundance, potentially leading to higher fitness of the kin^47^ and where the post-reproductive females are also known to increase adult male survival^8^. In elephants, the elders can be vast repositories of knowledge especially during harsh times^31^, and the experience and knowledge of the older generation seems highly beneficial to their descendants^27^,^30^.

In support of the knowledge of experienced females, we found that the more offspring the grandmother had raised herself before the grandcalf was born, the lower the mortality of the grandcalf. Thus, our results suggest that the grandmother’s experience in calf rearing might have been pivotal in enhancing grandcalf survival. That the grandmother presence is most beneficial for her young daughters’ calf survival further implies that the experience of the grandmother was crucial, whereas the older daughters had probably already learnt the needed mothering skills.

In comparison to humans, the effect sizes of grandmaternal effects in elephants are noteworthy. Hazard ratios of death for a grandchild in the absence of a grandmother have been below 3 during the most susceptible age in humans, in populations with clear grandmaternal advantages^48^ (and odds ratios and risk ratios below 3 have also been reported in other human populations^48^). Similarly, the grandmother shortened the inter-birth intervals in Asian elephants by almost a year, but in humans the decrease was around 3 months and only during the first three birth intervals^7^ (although humans have generally shorter average birth intervals than elephants). The grandmaternal effects discovered here resemble the effect size of mother presence on calf mortality in elephants during the first year of life^49^ (OR 10). In sum, compared to humans with menopause and long post-reproductive lifespan, our current study shows that grandmother effects can be even larger in magnitude in Asian elephant females that maintain reproductive function at least into their 60 s^3^.

Why then have some long-lived social species with inter-generational fitness benefits evolved menopause and others have not? Our results showing large positive effects of grandmothers on their daughter’s reproductive success irrespective of their own current reproductive status or age in Asian elephants imply that the grandmother effects are insufficient alone to generally result in the evolution of menopause^50^,^51^,^52^. In elephants, such benefits appear at least partly related to transferring experience to own young daughters, which does not require giving up own reproduction at advanced ages (menopause). However, these grandmother effects may play a part in the evolution of long (but not necessarily post-reproductive) lifespans according to the grandmother hypothesis. An alternative hypothesis for the evolution of menopause, the reproductive conflict hypothesis^53^ suggests that the costs and benefits of continuing to reproduce with age differ in long-lived species with differing dispersal patterns and age-specific changes in local relatedness^54^. Elephant herds consist usually of maternal relatives and thus relatedness in the group diminishes with age or stays the same, as males disperse from their natal group^35^,^55^. Instead, relatedness in resident killer whale pods increases with age as both sons and daughters stay in their natal group^56^, and this has been suggested to induce ageing females to shift their efforts from own reproduction to helping their existing descendants^56^. Similarly, in ancestral humans with a putative female-biased dispersal pattern, as a female gets older in her new group, she becomes also increasingly related to the rest of the group, which (at the face of any reproductive conflict with other group females) increases the benefits of helping instead of breeding herself. In such situations, the conflict arising from simultaneous intergenerational breeding might be too severe, affecting the cost-benefit ratio of continued breeding^50^,^53^. This conflict hypothesis should be further studied in other long-lived species such as elephants, to clarify the selection pressures on post-reproductive lifespan and long life in general, and on menopause evolution specifically.

Finally, the finding that multi-generational group composition enhances both calf survival and female birth rates is important for conservation issues concerning this endangered species as well as for population management. Calf survival is known to be one major limiting factor of elephant population growth with 25–50% calf mortality by age 5 in most studied populations, and particularly high calf mortality rates among zoo populations^35^,^36^,^57^,^58^. In this study calf survival was markedly higher for those in close proximity to their grandmother and thus these results provide conservationists and captive population managers with a potential way to boost their population by simply keeping the grandmothers with their (grand)offspring; in zoos multigenerational groups of elephants are rare and moving animals between zoos is common^59^. The results also further emphasize the need to prevent poaching targeting old, large females^60^ as their presence is crucial for the younger generation and whole group success, and removal of these individuals from the population might have severe outcomes for the species.

## Methods

### Study population

Myanmar has the largest population of captive elephants, estimated to be around 6000, and the majority of these elephants work in the timber industry^61^. We use a large multi-generational demographic dataset on this semi-captive Asian elephant population from Myanmar^3^,^49^,^57^,^62^,^63^. The dataset has been collected by Khyne U Mar from elephant logbooks and annual extraction reports archived and maintained by the Extraction Department, Myanma Timber Enterprise, and it covers the life-history of succeeding generations of captive timber elephants born into the population or captured from the wild for over a century. The dataset contains information on each individual of: registration number and name of elephant; origin; date and place of birth; mother’s registration number and name; capture method (if wild caught), age, year and place of capture; year or age of taming; dates and identities of all calves born; date of death or last known date alive; and cause of death. While the ages of captive-born elephants are known because precise birth dates are recorded, elephants caught from the wild are aged by comparing their height and body condition at the time of capture with captive elephants of known age.

The timber elephants of Myanmar live in forest camps, distributed across the country. Regulations are imposed on all elephants with set working hours per week, days per year and tonnage per individual. The elephants are employed during the day as riding, transport and draft animals and at night the elephants forage in the forest in their family groups unsupervised where they find food and encounter tame and wild conspecifics. Calves born in captivity are cared for by their biological and allo-mothers, and suckle until lactation no longer supports their demands. Working females are given rest from mid-pregnancy (11 months into gestation) until the calves reach their first birthday^64^. Mothers are then used for light duties but allowed to nurse the calves on demand; neither mothers nor calves have traditionally been supplemented with food by their human care-takers. The calves are generally weaned at the age of 4 or earlier when they are capable of independent foraging. They are separated from their mother and tamed around age 5 and then assigned a mahout, name, log-book and registration number and trained and used for light work as baggage elephants until age 17. The weaning and training age are known to present a challenge for calf survival^57^. By age 17, the elephants enter the work-force as full working elephants. They are retired at age 55^64^,^65^ and live out the rest of their lives in comparative freedom, but their logbooks are maintained until death.

From the large multi-generational dataset available, we extracted a subset of live-born calves, for which the grandmother was known to be alive at the time of their birth in captivity. For these calves we collected information on the specific working locations where the calves were born as well as where their mother and grandmother were present during that time. This resulted in 161 calves (F = 71, M = 90) from 95 mothers and 83 grandmothers. These calves were born during 1965–2000 and come from 29 logging locations (grandmothers from 32 locations) within 8 larger geographic regions (out of a total of 14) in Myanmar: Ayeyarwady, Bago, Chin, Kachin, Magway, Mandalay, Sagaing and Shan. All selected mothers and calves were captive-born with known birth date, but 87 of these calves had a grandmother which was captive born and 74 a grandmother with wild origin (and thus with estimated age only at the time of capture). There are no ethical issues related to the demographic data on Asian elephants because it is historical and no experimental protocols were used in collecting the dataset.

### Statistical analyses

All statistical analyses were conducted using SAS (SAS Institute Inc., release 9.3, 2002–2010). Final models were determined using backward elimination technique and terms dropped if they failed to reach statistical significance (*p* = 0.05) and when possible, we also used AIC criterion for model fit to the data.

All 161 live-born calves in our sample had a living grandmother at the time of their birth, with 112 having a grandmother living in a different logging location to themselves and 49 a grandmother living in the same location. The 49 calves which lived in the same location as their grandmother were born to 33 grandmothers (and 35 mothers), the grandmothers having 1–6 grandcalves in the same location. However, most grandmothers had only one daughter (*n* = 31) and one grandcalf (*n* = 26) alive in the same location. The 112 calves which lived in a different location to their grandmothers were born to 61 grandmothers (and 66 mothers), the grandmothers having 1–4 grandcalves in different locations to themselves. Of the 161 calves, 31 died before 5 years, 103 were successfully followed until age 5 (independence), and 27 were censored before age 5 due to being alive at the end of the study period. This is in line with the observed overall calf mortality in the population at around 25% of calves dying before age 5^57^, and also matches calf mortality rates in the wild^36^.

First we investigated whether the grandmother presence in the same location vs. another location at the time the grandcalf was born affected calf survival until age 5 by using Cox proportional hazards model. In this analysis all calves were included (*n* = 161) and the main term of interest was grandmaternal proximity in the same (*n* = 49) or another logging location (*n* = 112) compared to the grandcalf. We also controlled for calf sex, birth cohort (1 = 1965–1985, 2 = 1986–1993, 3 = 1994–2000) and larger geographic region (Mandalay, Sagaing, Bago, Magway, Kachin, Shan), birth order (1 = first born, 2 = 2nd–4th born), previous interbirth-interval (short = 1.80–5.16 years, long = 5.17–15.00 years, first born), maternal age (young = 10.50–20.30 years, old = 20.31–40.44 years), grandmaternal age (young = 25.7–44.63 years, old = 44.64–79.00 years), maternal survival (0–5 years; how long the mother survived after calf birth or if she was censored: surviving the entire 5 years equaled 5), grandmaternal survival (0–5 years, coded similarly as maternal survival) and grandmother’s origin (C = captive born, W = wild caught). Birth cohort is known to affect survival^57^ and was categorized according to the distribution and quartiles of the birth year (25%, 75%), whereas maternal and grandmaternal ages were categorized according to the mean (20.31 and 44.64 years, respectively) as was previous interbirth-interval (mean 5.17 years). These categories allowed meaningful interaction terms with the term of interest (grandmother presence) in order to test whether the grandmother effects differed between different time periods, between young and old mothers or grandmothers, or when reproduction was slow vs. fast. The grandmother identity was included as a random term in the model to adjust for repeated datapoints from the same individuals. When controlling for large-scale geographic variation in demographic rates across Myanmar, Chin and Kachin were grouped together because of low sample size and their location on mountainous regions. Similarly, Ayeyarwady was grouped with Bago because of low sample size and location on coastal regions. The mortality hazard was proportional for the grandmother proximity variable (χ_*1*_^2^ = 0.050, Adjusted *p* = 0.81), for the maternal age (χ_*1*_^2^ = 0.0050, Adjusted *p* = 0.94) and for the interaction between grandmother proximity and maternal age (χ_*1*_^2^ = 0.0018, Adjusted *p* = 0.96). The results of this analysis and the effect of the grandmother are corroborated by a more detailed analysis (Cox survival model with the same controlled variables as above) where we only included calves whose grandmother was known to be alive and in the same (*n* = 33) or another location (*n* = 75) until the calf died or reached age 5. This analysis confirmed the effect of the grandmaternal and maternal age being similar and significant (χ_*1*_^2^ = 4.34, Adjusted *p* = 0.023).

Second we investigated whether grandmother presence in the same location vs. another location at the time the grandcalf was born affected her daughter’s next birth interval length. This next birth interval was, on average, 4.97 ± 1.91 years (median = 4.6). Lastborn offspring and those offspring without siblings were excluded from this analysis. Elephant pregnancy lasts around 22 months and birth intervals <1.8 and >12.4 years (95% quartile of birth interval length) were therefore excluded as erroneous or outliers. Inter-birth intervals where the grandmother/ mother had changed location between the births of the first and second calf (e.g. grandmother was known to be in the same location at the birth of the first calf and in another location at the birth of the following calf or vice versa) were excluded to avoid contrasting grandmother effects (*n* = 5). Only birth intervals after live-born calves and during which the grandmother was known to be alive were included. This resulted in 75 birth intervals with the grandmother in the same (*n* = 17) or another location (*n* = 58). The 17 intervals (calves) during which the grandmother lived in the same location were from 10 grandmothers and 11 mothers and the 58 intervals during which the grandmother lived in a separate location were from 41 grandmothers and 42 mothers. We investigated the effect of grandmaternal proximity on inter-birth intervals using Glimmix procedure in SAS with gamma distribution and log-link. In this analysis Chin, Kachin and Shan were again grouped together when adjusting for large-scale variation across Myanmar because of low sample size and their location on mountainous regions. Also, Ayeyarwady and Mandalay were grouped with Bago because of low sample size and location on lowland regions. This resulted in a total of four geographic regions across Myanmar. Calf survival is known to affect inter-birth intervals^3^ and therefore we controlled for the survival of the first calf to age 2. Otherwise, the fixed terms and the random term, grandmother identity, were the same as in the above analysis. Non-significant mother identity nested within the grandmother identity as a random term was dropped from the final model (estimate ± S.E.: 0.026 ± 0.025, χ_*1*_^2^ = 1.09, p = 0.15).

Third we investigated how the grandmother’s reproductive status or previous number of calves at the time the grandcalf was born affected her grandcalf survival until age 5 by using Cox proportional hazards model. We only included calves with the grandmother in the same location (*n* = 49). The grandmother’s reproductive status was coded as whether the grandmother had given birth within 3 years of the birth of the grandoffspring (before or after) as this can be seen as the most dependent period of calf care^49^ and potentially has an effect on the amount of investment made by the grandmother in the grandoffspring. This resulted in 28 still reproducing grandmothers and 21 non-reproducing grandmothers. The number of calves born to the grandmother (before the grandoffspring) was coded as a continuous variable ranging from 2 to 7 calves (mean 4.47 ± 1.29 calves). The same controlled terms were considered as for the analysis of calf survival outlined above except for the regions across Myanmar, which were categorized similarly as in the inter-birth analysis into four categories. Non-significant grandmother identity as a random term was dropped from the final model (estimate ± S.E.: 0.14 ± 0.87, χ_*1*_^2^ = 0.97, Adjusted p = 0.30). The mortality hazard was proportional for the previous number of calves born to the grandmother (χ_*1*_^2^ = 0.30, *p* = 0.58), for the maternal age (χ_*1*_^2^ = 0.041, *p* = 0.84) and for the grandmother’s reproductive status variable (χ_*1*_^2^ = 0.0023, *p* = 0.96).

Finally we also tested whether being resident alongside at least one young (<20 years) daughter had an effect on the total number of grandoffspring born as compared to females which were not resident alongside any of their daughters, or were only resident alongside their older (>20) daughters. We investigated this by using Glimmix procedure in SAS with negative binomial distribution and log-link. In this analysis we used all grandmothers included in the grandcalf dataset, 83 grandmothers. Of these grandmothers 43 were known to be already dead, whereas 40 were still alive at the time of censoring, and this was controlled for in the analysis. Of these grandmothers, 25 were known to be in the same location with at least one young reproductive daughter and 58 were in another location or resident with only older daughters. The mean number of grandcalves for the grandmothers was 3.01 ± 1.98 (1–11). In this analysis Kachin and Shan were grouped together when adjusting for large-scale variation across Myanmar because of low sample size and their location on mountainous regions. Also, Mandalay was grouped with Bago because of low sample size and location on lowland regions. Other living areas in the analysis were Magway and Sagaing. We also controlled for birth cohort of the grandmother (1 = 1907–1932, 2 = 1933–1954, 3 = 1955–1969; categorized according to the quartiles of the birth year (25%, 75%)), grandmother’s origin (C = captive born, W = wild caught), age at first reproduction (mean 21.03, range 10.32–43.28), lifespan at death or at censoring (mean 52.73, range 34–82), number of reproductive daughters (1–2, only 3 had more than 2 and were grouped as having 2) and age at last reproduction (mean 42.63, range 21.52–68.25). Terms included in the final model were the residence near the <20 year old daughter, number of reproducing daughters Est. (1 reproducing daughter) = − 0.67 ± 0.15; (*F*_1,76_ = 19.42, p < 0.0001), origin Est. (captive born) = 0.30 ± 0.18; (*F*_1,76_ = 2.92, p = 0.098), lifespan Est. = − 0.0077 ± 0.0093; (*F*_1,76_ = 0.28, p = 0.60), whether the grandmother was still alive or not Est. (censored) = − 1.34 ± 0.74; (*F*_1,76_ = 3.27, p = 0.074), and interaction between lifespan and being alive or not Est. (censored) = 0.024 ± 0.014; (*F*_1,76_ = 2.81, p = 0.098). The results from this model should be interpreted with knowledge that the overall numbers of grandoffspring and the effects of lifespan on this outcome are under-estimates due to several females (and their daughters) still being alive and reproductively active.

## Additional Information

**How to cite this article**: Lahdenperä, M. *et al.* Nearby grandmother enhances calf survival and reproduction in Asian elephants. *Sci. Rep.* **6**, 27213; doi: 10.1038/srep27213 (2016).

## Figures and Tables

**Table 1:** Cox regression model of the effect of grandmaternal proximity on grandcalf mortality from birth to age 5 years.

**Term**	**Parameter estimate** **±** **S.E**	**Statistic (χ2)**	**DF**	**P value**	**Adjusted DF**	**Adjusted P value**
grandmother proximity (0 = another location)	−2.128 ± 1.022	4.332	1	0.037	0.765	0.025
calf sex (0 = female)	−0.872 ± 0.944	0.852	1	0.356	0.872	0.311
birth cohort (0 = 1986–1993)	higher later	8.390	2	0.015	1.509	0.008
grandmother’s origin (0 = captive born)	1.322 ± 0.855	2.396	1	0.122	0.721	0.080
maternal age (0 = young)	−2.092 ± 0.895	5.460	1	0.020	0.815	0.014
living region (0 = Magway)	random variation	12.247	5	0.032	3.399	0.010
grandmother proximity* maternal age	Fig 1a.	4.846	1	0.028	0.779	0.019
calf sex* maternal age		6.911	1	0.009	0.794	0.006
calf sex* grandmother origin		4.455	1	0.035	0.815	0.026
grandmother identity	1.61 ± 0.79	33.837			20.243	0.030
birth-order (0 = first born)	−0.155 ± 0.510	0.092	1	0.762	0.751	0.651
inter-birth interval		0.538	2	0.764	1.568	0.657
grandmaternal age (0 = young)	−0.093 ± 0.663	0.020	1	0.888	0.685	0.769
maternal survival	0.077 ± 0.240	0.102	1	0.749	0.795	0.659
grandmaternal survival	0.007 ± 0.197	0.001	1	0.972	0.865	0.954

Positive and negative estimates for the categorical variables mean that the mortality is higher and lower than in the reference group (0), respectively. Estimates are shown only for continuous and two level variables. For continuous variables, positive estimates indicate an increase in mortality risk, while negative estimates indicate a decreasing mortality function. N = 161 offspring. S.E. = standard errors. Terms retained in the final model are shown above the dashed line. Grandmother’s identity controlled as a random measure. All P-values are 2-tailed.

**Table 2:** Effect of grandmaternal proximity on daughter’s inter-birth intervals.

**Term**	**Parameter estimate ± S.E**	**Statistic (*****F***_**numdf, dendf**_)	**P value**
grandmother proximity (0 = another location)	−0.235 ± 0.090	6.73_1,68_	0.012
calf survival (0 = died)	0.243 ± 0.115	4.51_1,68_	0.037
calf sex (0 = female)	0.135 ± 0.076	3.19_1,68_	0.079
maternal age (0 = young)	0.308 ± 0.120	1.82_1,68_	0.182
calf birth-order (0 = first born)	−0.224 ± 0.110	0.11_1,68_	0.742
calf birth-order*maternal age		5.57_1,68_	0.021
grandmother’s origin (0 = captive born)	−0.004 ± 0.077	0.00_1,67_	0.960
birth cohort (0 = 1994–2000)	longest earlier	0.94_2,66_	0.397
living region (0 = Sagaing)	random variation	0.15_3,65_	0.930
grandmaternal age (0 = young)	−0.011 ± 0.083	0.02_1,67_	0.893

Positive and negative estimates for the categorical variables mean that the birth interval is longer and shorter than in the reference group (0), respectively. Estimates are shown only for continuous and two level variables. N = 75 offspring/birth-intervals. S.E. = standard errors. Terms retained in the final model are shown above the dashed line. All P-values are 2-tailed.

**Table 3:** Cox regression model of the effect of grandmother’s reproductive status and previous number of calves born on grandcalf mortality from birth to age 5 years.

**Term**	**Parameter estimate ± S.E**	**Statistic (χ^2^)**	**DF**	**P value**
previous number of calves of gm	−0.84 ± 0.38	4.81	1	0.028
maternal age (0 = young)	3.01 ± 1.03	8.61	1	0.0034
gm reproductive status (0 = reproducing)	0.53 ± 0.76	0.49	1	0.48
calf sex (0 = female)	−0.33 ± 0.74	0.20	1	0.65
birth cohort (0 = 1986–1993)	higher later	4.39	2	0.11
grandmother’s origin (0 = captive born)	−0.42 ± 0.75	0.31	1	0.58
living region (0 = Sagaing)	random variation	4.31	3	0.23
calf birth-order (0 = first born)	0.065 ± 0.79	0.0067	1	0.93
inter-birth interval		1.30	2	0.52
grandmaternal age (0 = young)	−0.58 ± 0.73	0.63	1	0.43
maternal survival	−0.068 ± 0.23	0.084	1	0.77
grandmaternal survival	−0.071 ± 0.23	0.092	1	0.76

Positive and negative estimates for the categorical variables mean that the mortality is higher and lower than in the reference group (0), respectively. Estimates are shown only for continuous and two level variables. For continuous variables, positive estimates indicate an increase in mortality risk, while negative estimates indicate a decreasing mortality function. N = 49 offspring. S.E. = standard errors. Terms retained in the final model are shown above the dashed line. Gm refers to grandmother. All P-values are 2-tailed.
